# Synthesis and Catalytic Degradation of PEF, ENR, and CIP by g-C_3_N_4_/TCNQ/Eu Composite

**DOI:** 10.3390/mi14122146

**Published:** 2023-11-24

**Authors:** Hongyue Chen, Jianxin Geng, Jinghui Shen, Qi Shi, Jingxue Lv, Yuguang Lv, Chaoyu Song

**Affiliations:** 1 College of Pharmacy, Jiamusi University, Jiamusi 154007, China; 2 School of Chemistry and Chemical Engineering, Shanghai Jiao Tong University, Shanghai 200240, China

**Keywords:** antibiotics, photocatalytic degradation, degradation pathway

## Abstract

By using melamine as a precursor for the copolymerization process, g-C_3_N_4_ and g-C_3_N_4_/TCNQ/Eu complexes with various amounts of doping were created. These complexes were then examined using XRD, FT-IR, SEM, TEM, XPS, PL, UV-vis, and I-T. The degradation rates of pefloxacin (PEF), enrofloxacin (ENR), and ciprofloxacin (CIP) were 91.1%, 90.8%, and 93.2% under visible light (λ > 550 nm). The photocatalytic performance of the composite was analyzed, and the best effect was obtained for CIP photocatalysis when Eu doping was 3 mg at 20 °C and pH 7. Kinetic analysis showed that there was a linear relationship between the sample and the photocatalytic time, and the degradation rate was about 5 times that of g-C_3_N_4_. The cyclic stability of the g-C_3_N_4_/TCNQ/Eu composite sample was found to be good through repeated experiments. UPLC-MS visualizes the degradation process of CIP. The extremely low stability of piperazine ring induced subsequent degradation, followed by the fracture of quinolone ring promoting the complete decomposition of CIP.

## 1. Introduce

In recent years, with the acceleration of the economic and social development of our country, the sources of environmental pollution have expanded from common persistent organic pollutants to drugs with biochemical activity [1]. Antibiotics, as a class of widely used antibacterial drugs, are mainly used to treat various diseases caused by non-viral infections in humans and animals. Among them, quinolones (QNs), as the second most used antibacterial drugs, are widely used in medical and veterinary clinical medicine [2]. Microbial resistance caused by the abuse of quinolone antibiotics has posed a threat to the ecological environment and human health [3,4]. Traditional water treatment processes have been unable to effectively remove quinolone antibiotics from medical industrial wastewater, animal feed wastewater, aquaculture wastewater, and domestic sewage [5]. Therefore, the selection of a new efficient, green, and environmentally friendly degradation method for quinolones is the key to solve the pollution problem of quinolones in the environment, and it is also a hotspot of current research [6,7]. Studies have found that semiconductor photocatalytic oxidation technology is an effective means to use solar energy to degrade pollutants in the environment. In solving the problem of antibiotic pollution, it has great advantages such as energy saving, high efficiency, and thorough degradation of pollutants [8,9,10,11].

As a new type of metal-free polymer semiconductor, g-C_3_N_4_ has the advantages of low costs and stable performance, and is a semiconductor photocatalytic material with a good visible light response [12,13]. Like other compounds, when a g-C_3_N_4_ monomer is applied to photocatalysis, the photocatalytic efficiency of g-C_3_N_4_ is not ideal due to some serious defects of g-C_3_N_4_ itself [14]. Mainly due to the poor excitation effect of photogenerated charge carriers, small specific surface area, fewer active sites, the separation and transmission of photogenerated electrons is not ideal, and the absorption capacity of visible light is poor. Therefore, it is particularly important to modify and optimize g-C_3_N_4_ [15,16,17,18,19].

Metal doping has been proven to be a significant method for g-C_3_N_4_ modification. This type of method can adjust the potential of conduction and valence bands, enhance light absorption ability, and significantly enhance the photodegradation performance of g-C_3_N_4_ [20,21,22]. Doping lanthanides in semiconductors helps to regulate the performance of semiconductors to a greater extent. The unique 4f electronic configuration and spectral characteristics enable lanthanide as a dopant to change the crystal structure, optical properties, and surface adsorption of g-C_3_N_4_ [23]. That is, the modification of g-C_3_N_4_ by doping rare earth metals can reduce the band gap, effectively prevent the photogenerated electron-hole pair recombination, and expand the response spectrum range, thereby enhancing the photochemical activity of the catalyst. Eu is the most active rare earth element. Previous studies have proved that doping Eu element can effectively improve the light absorption of g-C_3_N_4_, extend it to the visible light range, and promote the separation of photogenerated electron-hole pairs, thus maximizing the efficiency of photocatalytic reaction [24,25,26]. The organic superconductor 7, 7, 8, 8-tetracyanop-benzodiquinone dimethane (TCNQ) is a complex salt and has excellent electrical conductivity properties [27,28,29,30,31,32].

In this study, we synthesized a new photocatalyst by doping together g-C_3_N_4_, TCNQ, and Eu elements for the first time. The catalyst showed remarkable photocatalytic effect on PEF, ENR, and CIP. The enhanced effect of Eu and TCNQ on the light response of g-C_3_N_4_ has been confirmed. However, there has been no molecular level analysis of its degradation process. Therefore, this study qualitatively analyzed the intermediates in the degradation process through UPLC-MS and speculated on a possible degradation pathway. The visualization of the degradation process makes the decomposition of CIP easier to understand.

## 2. Experiment

### 2.1. Composite Material Synthesis

A total of 4 g of melamine and 20 mg of TCNQ were mixed and ground evenly before being collected in a crucible. A certain amount of Eu_2_O_3_ powder was dissolved in 15 mL of dilute nitric acid (20%), and then poured into the crucible and stirred to obtain a light green paste solid. The prepared solid mixture was put into a muffle furnace and heated to 550 °C for 4 h. After cooling to room temperature, it was removed from the mufter, and the obtained g-C_3_N_4_/TCNQ/Eu-x sample was ground into powder for later use, where x represents the amount of Eu added (x = 1 mg, 2 mg, 3 mg, 4 mg).

### 2.2. Experiments on the Catalytic Degradation of PEF, ENR and CIP by g-C_3_N_4_/TCNQ/Eu Composite

The PEF, ENR, and CIP standards were, respectively, weighed at 2.5 mg and ultrasonic dispersed in a bit of ethanol, then the volume was fixed to 250 mL. A total of 10 mg/L PEF, ENR, and CIP stock solution was obtained, stored in the dark, and diluted to the desired concentration with time.

Precisely weighed, 50 mg of the g-C_3_N_4_/TCNQ/Eu complex with various doping ratios were dissolved in 50 mL of various drug solutions at a concentration of 10 mg/L. The materials were first uniformly dispersed under ultrasound for 30 min, followed by 30 min of stirring with magnetic force in a dark atmosphere. After that, a 300 W xenon light was turned on to mimic an experiment where photocatalytic degradation had place. Every 30 min, 5 mL of the reaction solution were removed, and the supernatant was removed by centrifugation twice at a very fast speed. The absorbance at characteristic absorption wavelength was determined by UV-vis spectrophotometer(Hitachi, Tokyo, Japan). In order to avoid the effect of temperature change on the experimental results, the water cycle temperature should be maintained at about 20 °C.

### 2.3. Identification of Intermediate Products

Each time, the point’s sample solution was run through a 0.45 μm microporous filter membrane. Thermo Scientific Q Exactive Series mass spectrometer (Thermo Scientific, Waltham, MA, USA) and a Dionex Ultimate 3000 ultra-high performance liquid chromatograph (Thermo Scientific, USA) were used to measure the specific concentration of PEF, ENR, and CIP in each sample.

### 2.4. Conditions for Chromatographic Analysis and Mass Spectrometry

Conditions for mass spectrometry detection include the following: the ion source used was a multi-reaction monitoring mode (MRM) electron spray ion source (ESI); 40 psi spray pressure; 4000 volts in the capillary 12 L/min for the flow of atomized gas; temperature for drying was 350 °C.

Agilent Zorbax SB-C18 column (2.1 mm × 100 mm, 1.8 μm); column temperature: 30 °C; flow rate: 0.4 mL/min; 0.2% solution of formic acid and acetonitrile make up the mobile phase.

## 3. Result and Analysis

### 3.1. Characterization Results Analysis of g-C_3_N_4_/TCNQ/Eu

#### 3.1.1. X-ray Diffractometer

**Figure 1:**
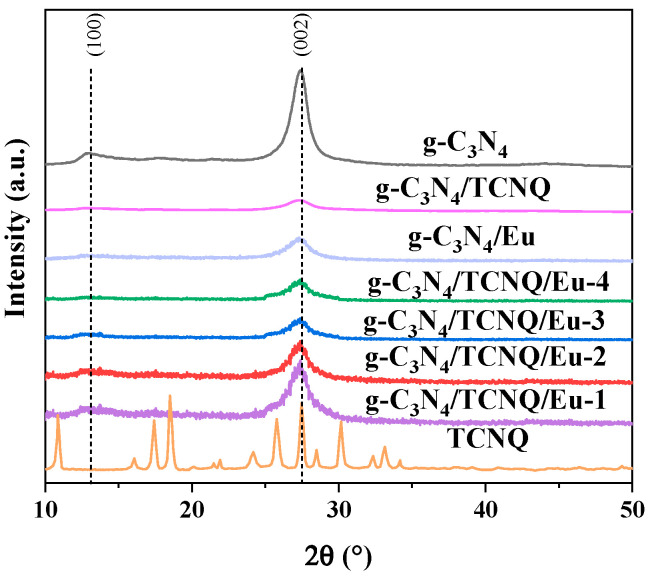
The XRD diffraction profiles of the g-C_3_N_4_, TCNQ, and g-C_3_N_4_/TCNQ/Eu composites.

The XRD patterns of the g-C_3_N_4_ and g-C_3_N_4_/TCNQ/Eu samples are shown in Figure 1. The graphic shows that the two typical peaks are located at 13.0° and 27.4°, respectively, which match the (100) and (002) crystal planes of g-C_3_N_4_. The (002) crystal plane of g-C_3_N_4_ represents the interlayer distance, and the (100) crystal plane represents the heptazine unit of g-C_3_N_4_, which is consistent with the crystal structure of g-C_3_N_4_ and highly consistent with the standard card ((JCPDS No. 87-1526)) [24,33]. The diffraction peak located at 27.4° is attributed to the stacking of crystal planes of the aromatic system, while the diffraction peak at 13.0° is attributed to the stacking of planar structures of 3-S-triazine, which is consistent with the crystal structure of g-C_3_N_4_. The crystal phase of g-C_3_N_4_ did not change with the increase in TCNQ content. Crystalline TCNQ appears only when its load exceeds the threshold [34]. The introduction of Eu did not change the locations and the number of peaks, but widened the peak width slightly, which indicated that Eu reduced the crystalline phase of g-C_3_N_4_ and increased the graphitization degree of g-C_3_N_4_ [35]. As a result, the g-C_3_N_4_/TCNQ/Eu sample can still exhibit the in-plane and inter-layer properties of g-C_3_N_4_, proving that the g-C_3_N_4_ with a structure such as graphene was created using a straightforward thermal copolymerization technique.

#### 3.1.2. Fourier Transform Infrared Spectroscopy

**Figure 2:**
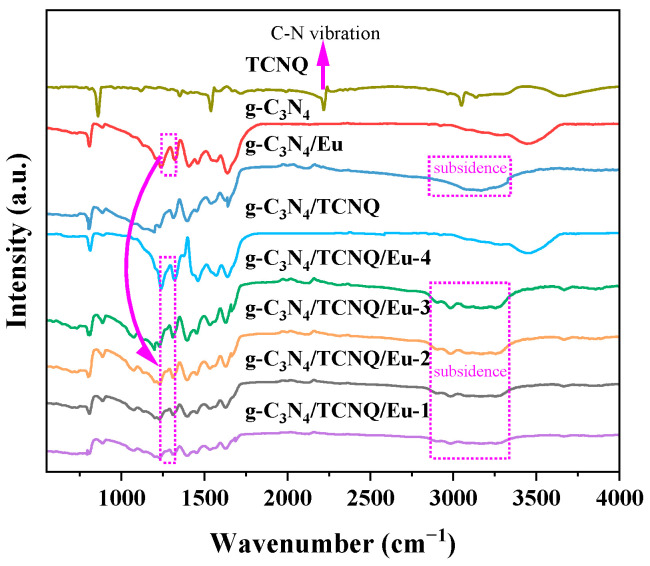
FT-IR profiles of g-C_3_N_4_, TCNQ, g-C_3_N_4_/TCNQ, g-C_3_N_4_/Eu, and g-C_3_N_4_/TCNQ/Eu composites.

Figure 2 shows the infrared spectrum of sample g-C_3_N_4_/TCNQ/Eu. The characteristic resonance peak of the aromatic C-N hetercyclic repeat unit in g-C_3_N_4_ is an absorption band in the 1200–1700 cm^−1^ range. C-NH-C, C-N, and C=N bonds are represented by the peaks at 1240, 1318, and 1573 cm^−1^ in this range, respectively [36,37]. At 810 cm^−1^, it corresponds to the 3-S-triazine ring (C_3_N_3_) structure [38]. In addition, the peak value of the N-H bond is 3000 to 3500 cm^−1^ [39]. The significant bands in the 2900 to 3400 cm^−1^ region originate from the stretching vibrational modes of the N-H and hydroxyl groups adsorbed to H_2_O. The broad peak group between 1200 and 1700 cm^−1^ is characteristic of the representative stretching pattern of the aromatic CN heterring. The peak at 805 cm^−1^ is attributed to the vibration mode of the 3-S-triazine unit. After doping TCNQ, the two peaks at 1292 cm^−1^ bifurcated. Small peaks appear at 1900–2500 cm^−1^. The C-N vibration peak of TCNQ can be clearly seen at 2223 cm^−1^ [40]. Compared to undoped samples, Eu caused subsidence at 2800 to 3300 locations. In addition, the FT-IR peak intensity decreased with increasing Eu doping, indicating that the g-C_3_N_4_ skeleton was partially altered by Eu doping and part of the triazine ring was destroyed. Eu doping does not change the structure of g-C_3_N_4_ although the adsorption position and strength are reduced.

#### 3.1.3. Scanning Electron Microscope

**Figure 3:**
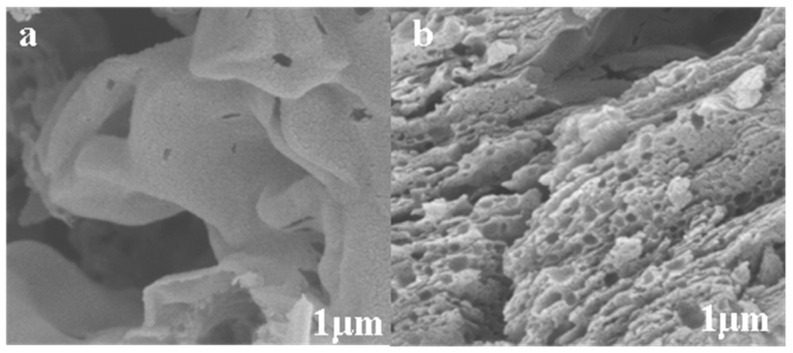
The SEM plot of g-C_3_N_4_ (**a**) and g-C_3_N_4_/TCNQ/Eu-3 (**b**).

The SEM diagram of the g-C_3_N_4_ and g-C_3_N_4_/TCNQ/Eu-3 samples is shown in Figure 3. It can be seen that large, thin sheets make up the structure of g-C_3_N_4_, but due to the doping of TCNQ and Eu, the sheet structure of g-C_3_N_4_ disappears, a large number of micropores are generated on the surface, and the size becomes smaller and smaller, forming a hollow lantern-like structure with a larger surface area. This greatly increases the absorption and response of the material to light. The findings demonstrate that the surface morphology of g-C_3_N_4_ can be dramatically altered by the copolymerization of TCNQ and Eu, leading to the formation of a hollow structure.

#### 3.1.4. Transmission Electron Microscope

**Figure 4:**
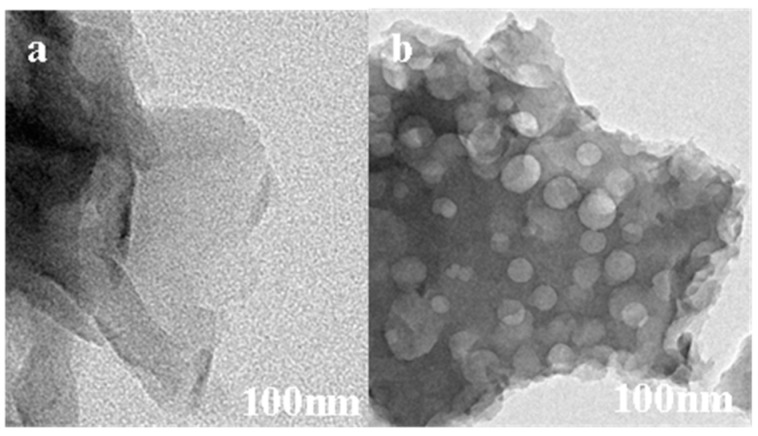
The TEM diagram of g-C_3_N_4_ (**a**) and g-C_3_N_4_/TCNQ/Eu-3 (**b**).

The TEM diagram of g-C_3_N_4_ and g-C_3_N_4_/TCNQ/Eu-3 is shown in Figure 4. The figure shows that all of the samples are two-dimensional nanomaterials. The original g-C_3_N_4_ two-dimensional sheet nanosheet has a large size, smooth surface, and complete structure, but the doping of TCNQ and Eu content causes the size of the g-C_3_N_4_ nanosheet to decrease and more hollow structures to form on the surface. It demonstrates how the splitting of nanosheets during the TCNQ and Eu copolymerization process can produce pores on the surface of g-C_3_N_4_ and increase the photocatalytic activity.

#### 3.1.5. X-ray Photoelectric Energy Spectrum

**Figure 5:**
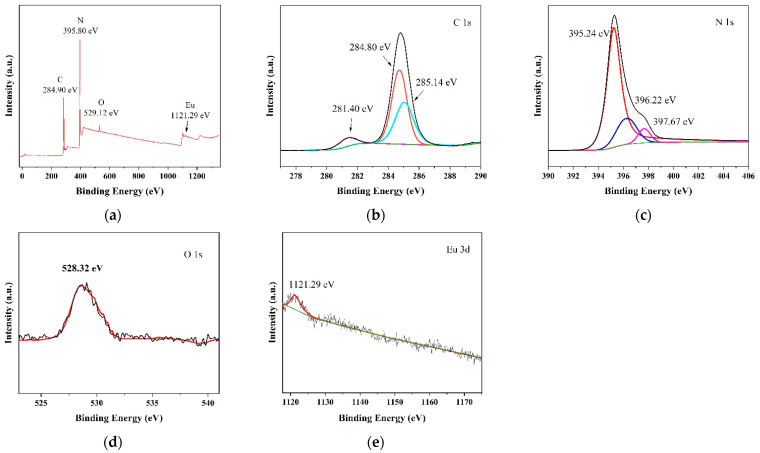
XPS patterns of g-C3N4/TCNQ/Eu-3 samples: (**a**) full spectrum, (**b**) C 1s, (**c**) N 1s, (**d**) O 1s, and (**e**) Eu 3d.

Figure 5 shows the XPS spectra of g-C_3_N_4_/TCNQ/Eu-3 samples. The figure shows that there are four elements O, C, N, and Eu with sharp peaks at the binding energies of 284.8 eV and 395.2 eV, which are attributed to C 1s and N 1s, respectively. The spectra in the high resolution case of C 1s are at 288.15 eV, 287.47 eV, and 284.81 eV, corresponding to C−N=C, C−(N)_3_, and C−C bonds. For N 1s spectra, 395.2 eV is the N in sp^2^ hybrid C=N−C, 396.2 eV is the N in tertiary nitrogen group N−(C)_3_ bond, and 397.2 eV is the N in H−N−C, respectively. The O 1s peak at 528.3 eV shown can be assigned to the O element in g-C_3_N_4_/Eu_2_O_3_. Meanwhile, the O 1s peak of g-C_3_N_4_ at 528.3 eV is also from the hydroxyl group. The peak located at 1121.3 eV corresponds to Eu(3d) [41], which further proves the successful preparation of g-C_3_N_4_/TCNQ/Eu-3 composite samples through element type analysis.

#### 3.1.6. Photoluminescence (PL) Spectrum

**Figure 6:**
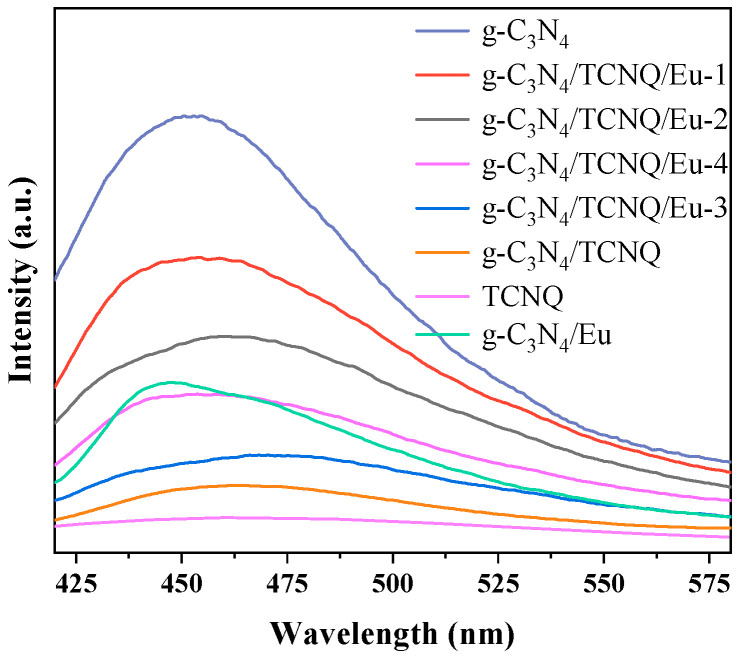
PL plot of g-C_3_N_4_, TCNQ, g-C_3_N_4_/TCNQ, and g-C_3_N_4_/TCNQ/Eu composites.

Both g-C_3_N_4_ and g-C_3_N_4_/TCNQ/Eu samples exhibit broad emission peaks about 460 nm, which is the typical band PL phenomena of g-C_3_N_4_ photoexcited charge carriers, as shown in Figure 6′s PL spectra. Different g-C_3_N_4_/TCNQ/Eu samples had lesser peak intensities than the original g-C_3_N_4_ due to doping. The results show that the separation and transmission efficiency of photoexcited charge carriers is significantly improved after doping TCNQ and Eu, and the doping of empty spheres and Eu is beneficial to prevent the radiation recombination of photoexcited electrons and holes. In addition, the introduction of Eu will cause the emission peak of g-C_3_N_4_ to shift significantly to around 450 nm. The hollow structure has a good solar reflection structure, which is favorable for light absorption, and is beneficial for charge transport, thus further inhibiting their recombination.

#### 3.1.7. UV-Vis Diffuse Reflectance Spectroscopy

**Figure 7:**
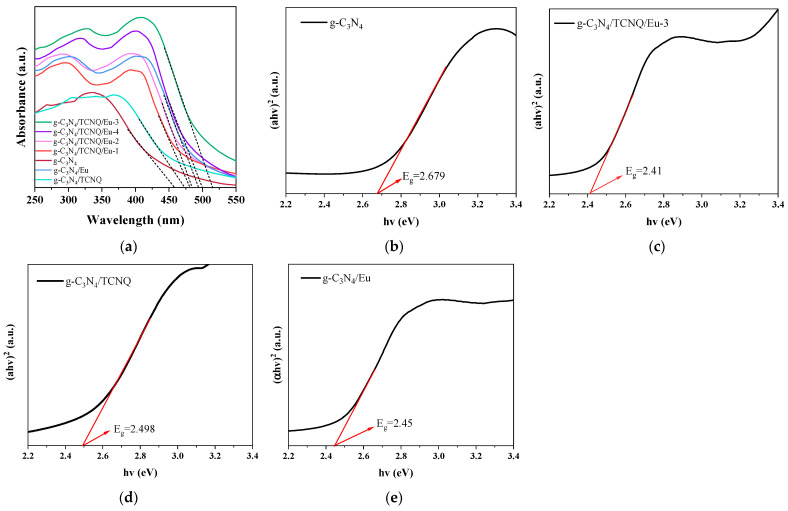
(**a**) UV-Vis diffuse reflectance spectra of g-C_3_N_4_/TCNQ/Eu-1, g-C_3_N_4_/TCNQ/Eu-2, g-C_3_N_4_/TCNQ/Eu-3, g-C_3_N_4_/TCNQ/Eu-4, g-C_3_N_4_/TCNQ, g-C_3_N_4_/Eu, g-C_3_N_4_ and (αhν)^2^ versus energy (hν) for the band gap energies of the samples: (**b**) g-C_3_N_4_, (**c**) g-C_3_N_4_/TCNQ/Eu-3, (**d**) g-C_3_N_4_/TCNQ, (**e**) g-C_3_N_4_/Eu.

The DRS plots of the g-C_3_N_4_ and g-C_3_N_4_/TCNQ/Eu samples are displayed in Figure 7. It can be observed from the figure that the absorption threshold for the g-C_3_N_4_ sample is at 462 nm, indicating a bandgap width of 2.68 eV. G-C_3_N_4_/TCNQ/Eu-1, g-C_3_N_4_/TCNQ/Eu-2, g-C_3_N_4_/TCNQ/Eu-3, g-C_3_N_4_/TCNQ/Eu-4, g-C_3_N_4_/TCNQ, and g-C_3_N_4_/Eu had absorption thresholds of 482 nm, 487 nm, 515 nm, 501 nm, 472 nm, and 494 nm, respectively. In addition to being smaller, the equivalent bandgap widths are 2.57 eV, 2.55 eV, 2.41 eV, 2.48 eV, 2.498 eV, and 2.45 eV, respectively. The findings demonstrate that the g-C_3_N_4_/TCNQ/Eu sample has a high visible light response capability, which is advantageous for the enhancement of photocatalytic performance.

#### 3.1.8. Photocurrent Test

**Figure 8:**
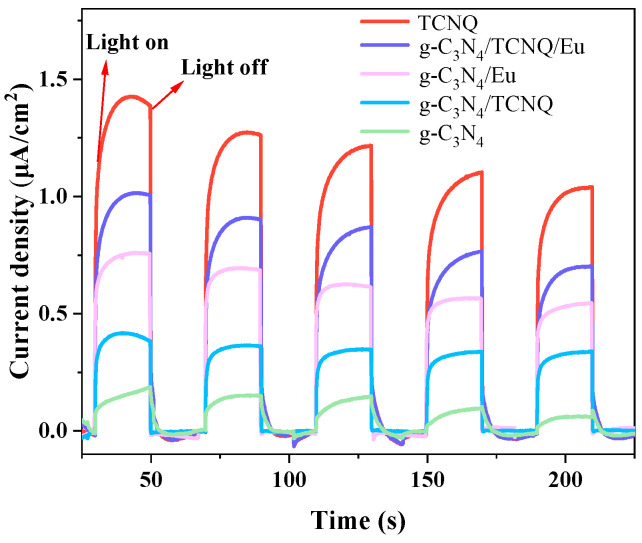
Responses of the g-C_3_N_4_ and g-C_3_N_4_/TCNQ/Eu composites to transient photocurrent.

Figure 8 display the I-T graphs of these four samples, which demonstrate how the photogenerated current rapidly falls when the light source is turned off while peaking when the light source is turned on. It shows that the current generation is caused by the photogenerated electron-hole in the material. G-C_3_N_4_/TCNQ/Eu > g-C_3_N_4_/Eu > g-C_3_N_4_/TCNQ > g-C_3_N_4_. The increased photocurrent intensity can be attributed to the favorable separation of photogenerated electron-hole by TCNQ and Eu copolymer doping, which can generate more charge carriers and thus benefit the improvement of photocatalytic activity.

### 3.2. Photocatalytic Performance Analysis of g-C_3_N_4_/TCNQ/Eu Composite

#### 3.2.1. Different Medications’ Effects on the Catalytic Effect

**Figure 9:**
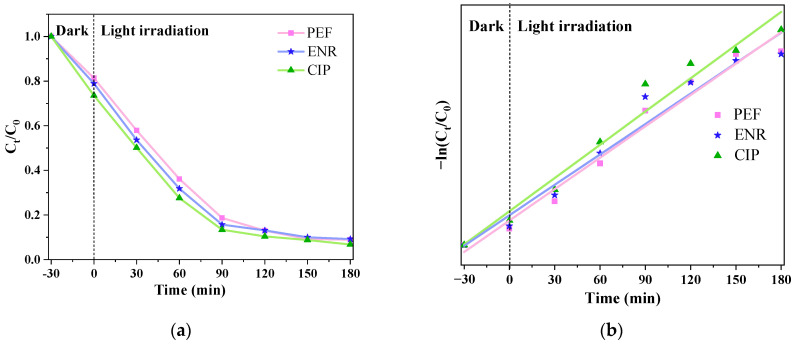
(**a**) The catalytic degradation of various drug combinations by the g-C_3_N_4_/TCNQ/Eu composite, and (**b**) the first-order reaction kinetic curves for various photocatalysts.

Figure 9a shows that when the samples doped with g-C_3_N_4_/TCNQ/Eu-3 are added to PEF, ENR, and CIP solutions, respectively. The dark reaction was carried out for 30 min, the dark reaction was complete, and the light reaction’s initial state was set to 0. D_PEF_ = 91.1%, D_ENR_ = 90.8%, and D_CIP_ = 93.2% were the results of a 180 min photocatalytic process. Studies reveal that medication ENR, CIP, and PEF samples had the best catalytic activity and a very high rate of degradation. The first-order kinetic technique was utilized to match the photocatalytic degradation kinetics of PEF, ENR, and CIP. As shown in Figure 9b, there was a good linear relationship between –ln(C_0_/C_t_) of each drug and the photocatalytic reaction time t.

#### 3.2.2. Different Amounts of Copolymer Doping’s Effects on CIP’s Catalytic Degradation

**Figure 10:**
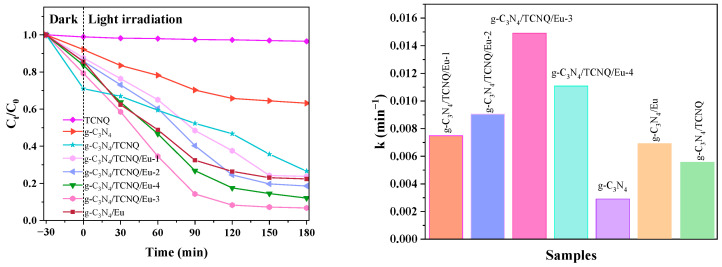
Degradation effects of g-C_3_N_4_ and g-C_3_N_4_/TCNQ/Eu composites with different Eu contents.

It can be seen from the Figure 10 that after 30 min of dark reaction, g-C_3_N_4_ has very low photocatalytic activity with D_ENR_ of 37.3% as time increases. Although g-C_3_N_4_ can efficiently absorb visible light, due to its tiny specific surface area, it is unable to perform photocatalysis. The D_CIP_ of g-C_3_N_4_/TCNQ/Eu-1 samples was 76.2%, the D_CIP_ of g-C_3_N_4_/TCNQ/Eu-2 samples was 81.4%, and the D_CIP_ of g-C_3_N_4_/TCNQ/Eu-3 samples was 93.2%. The D_CIP_ of g-C_3_N_4_/TCNQ/Eu-4 samples was 87.9%. Experiments show that within a certain doping range, Eu may enhance photocatalytic activity and help the photocatalytic process by being added in the right amount. When the g-C_3_N_4_/TCNQ/Eu-3 doping level is high, the degradation rate of CIP is up to 93.2%, which has the best catalytic activity.

#### 3.2.3. Different pH Levels’ Effects on the Catalytic Impact of CIP Degradation

**Figure 11:**
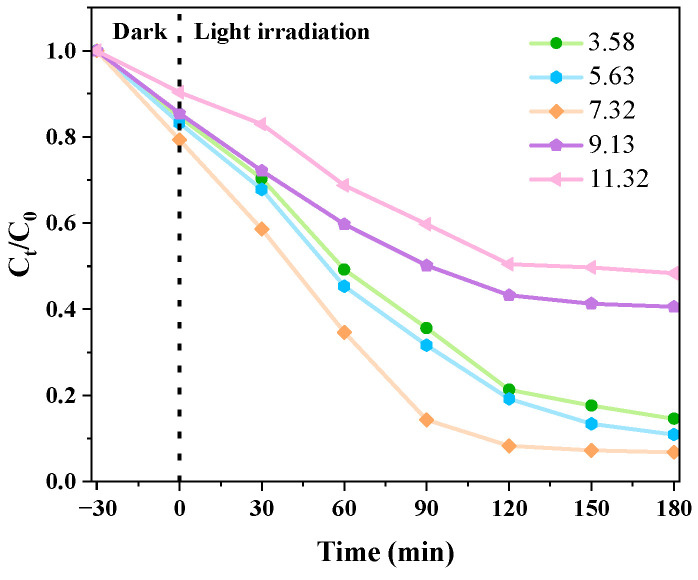
Effect of pH change on catalytic degradation of CIP in g-C_3_N_4_/TCNQ/Eu composites.

Figure 11 shows the CIP degradation rate of g-C_3_N_4_/TCNQ/Eu-3 under different pH values. The figure shows that when alkaline (pH = 11), g-C_3_N_4_/TCNQ/EU-3 degradation efficiency is greatly decreased, and the degradation rate of CIP is decreased compared with that of neutral environment. It may be due to the mutual repulsion between CIP and catalyst molecules under alkaline conditions, and the adsorption capacity of photocatalyst to pollutants is reduced, resulting in a weakening of degradation efficiency [42]. According to the mechanism of degradation of antibiotics by g-C_3_N_4_-based photocatalyst in reference [43], excessive OH^−^ will inhibit the generation of H_2_O_2_ from O_2_^−^ in the previous process, thus affecting the generation of hydroxyl radical, inhibiting the reaction to a certain extent, and reducing the catalytic activity of the reaction. At moderate acidity pH = 3 and weak acidity pH = 5, the degradation rate also showed a decreasing trend, but the trend was not obvious. The results show that the reaction is inhibited and the activity of the catalyst is affected under both acidic and basic conditions, and the influence of acidic condition is small. Therefore, the optimal pH of the solution during the photocatalytic degradation of CIP was 7.

#### 3.2.4. Effects of Different Temperatures on Catalytic Degradation of CIP

**Figure 12:**
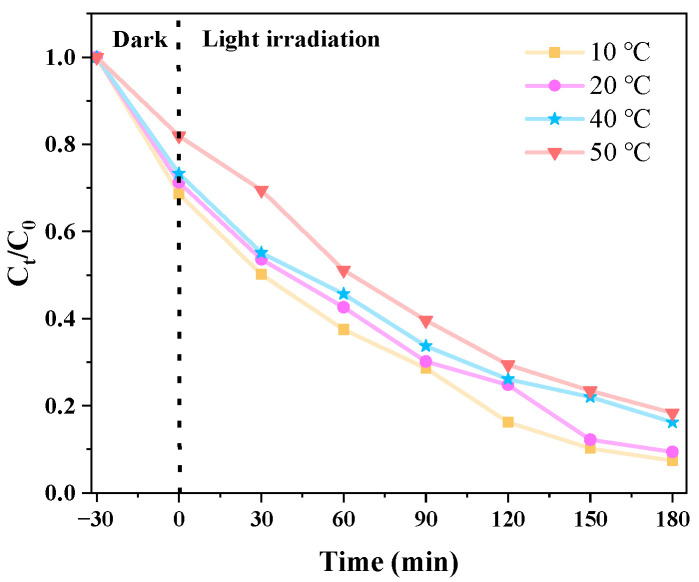
Effect of temperature changes on the catalytic activity of the g-C_3_N_4_/TCNQ/Eu composites.

Figure 12 shows the photocatalytic degradation effect of g-C_3_N_4_/TCNQ/Eu-3 at different reaction temperatures (10 °C, 20 °C, 40 °C, 50 °C) when the dosage of g-C_3_N_4_/TCNQ/Eu-3 is 50 mg and the concentration of CIP is 10 mg/L. It can be seen from the figure that the photocatalytic rate decreases with the increase in temperature under certain conditions. The degradation rates are 92.6%, 90.6%, 83.8%, and 81.7%, respectively. Since the activation energy of the semiconductor photocatalytic reaction is relatively low, the influence of the temperature of the reaction system on the photocatalytic reaction is mainly reflected in the impact on the collision frequency of the reaction molecules, adsorption equilibrium, and surface migration. Therefore, the temperature will affect the dark reaction phase of the reaction system to a certain extent. Relatively speaking, it is beneficial for adsorption at lower temperatures and desorption at higher temperatures. For this reason, 20 °C was used as the reaction temperature in this test.

### 3.3. Kinetic Analysis of Photocatalytic Degradation of CIP

**Figure 13:**
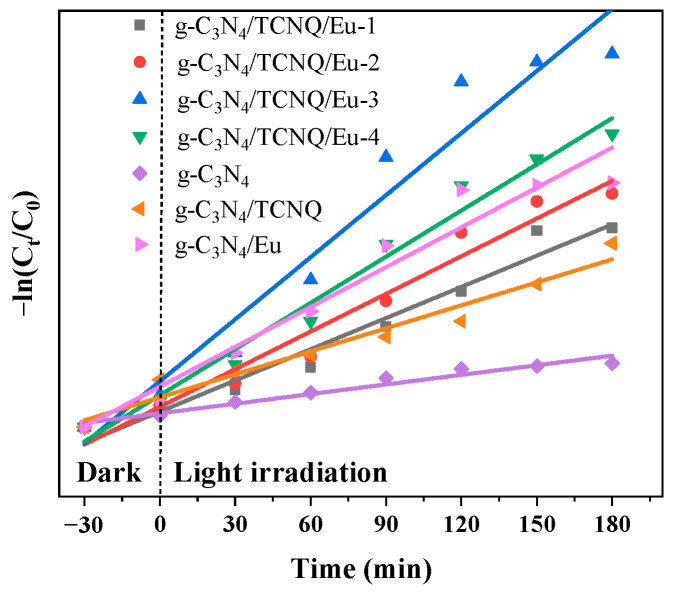
First-order reaction constants of g-C_3_N_4_/TCNQ/Eu photocatalytic reduction in CIP.

Figure 13 shows the kinetic evaluation of prepared g-C_3_N_4_/TCNQ/Eu’s photocatalytic degradation of CIP. To create an adsorption-desorption equilibrium, the composite samples were subjected to a 30 min dark reaction in CIP. After setting the end time of the dark reaction and the start time of the light reaction to zero, the kinetics of the photocatalytic reaction was investigated. The relationship between −ln(C_0_/C_t_) and the photocatalytic reaction time t for each sample can be shown to be linear in the figure.

According to Table 1, which is almost five times that of g-C_3_N_4_, g-C_3_N_4_/TCNQ/Eu-3 has the highest degree of CIP degradation and a higher k value, k = 0.0149 min^−1^. Each g-C_3_N_4_/TCNQ/Eu composite sample has an R^2^ that is greater than 0.9, indicating that each sample satisfies the agreement between the first-order reaction kinetic equation and the D_CIP_.

### 3.4. Analysis of Reuse Experimental Results of CIP Degradation by Catalyst

**Figure 14:**
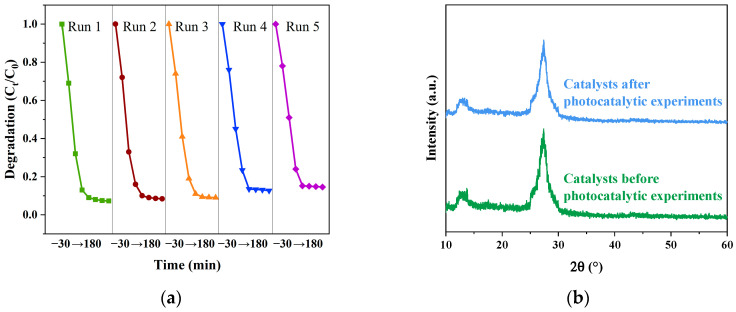
(**a**) Reuse experiment of g-C_3_N_4_/TCNQ/Eu composites degrading CIP and (**b**) XRD pattern of the catalyst (g-C_3_N_4_/TCNQ/Eu-3) after the reuse experiment and before the experiment.

Figure 14 shows the stable cycle diagram of the catalyst. The samples with the best photocatalytic performance are selected, filtered, sonicated, dried, recycled, and reused. The catalyst continued to degrade at a high rate of roughly 85% even after the experiment was repeated four times. The first degradation effect was the best, and the degradation rate dropped each time, but the difference was not significant. The findings demonstrate the catalyst’s good stability, reusability, and renewable performance. Moreover, the XRD pattern of the catalyst (g-C_3_N_4_/TCNQ/Eu-3) after the reuse experiment showed no significant difference from the pattern before the experiment, indicating the significant stability of the catalyst.

### 3.5. Analysis of Free Radical Capture Experimental Data of Catalyst-Mediated CIP Degradation

**Figure 15:**
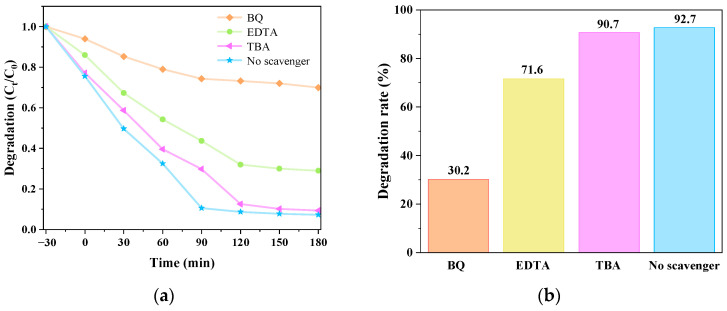
(**a**) Various radical capture agents’ effects on catalytic degradation and (**b**) degradation rate.

As shown in Figure 15, g-C_3_N_4_/TCNQ/Eu-3 can degrade CIP up to 92.7% without any capture agent. When BQ (p-benzoquinone), TPA (terylene acid), and EDTA (ethylenediamine tetraacetic acid) were added, the degradation rate decreased to 30.2%, 90.7%, and 71.6%, respectively. The amount of influence that various active ingredients have on the CIP degradation process’s outcomes corresponds to O_2_^−^ > h^+^ > OH. When BQ capture agent was added, the degradation rate of CIP was significantly reduced, showing that O_2_ and h^+^ were crucial components of the catalytic degradation mechanism, and ·O_2_^−^ was the most dominant active species.

### 3.6. Identification of Intermediate Products of CIP Degradation by Catalyst

**Figure 16:**
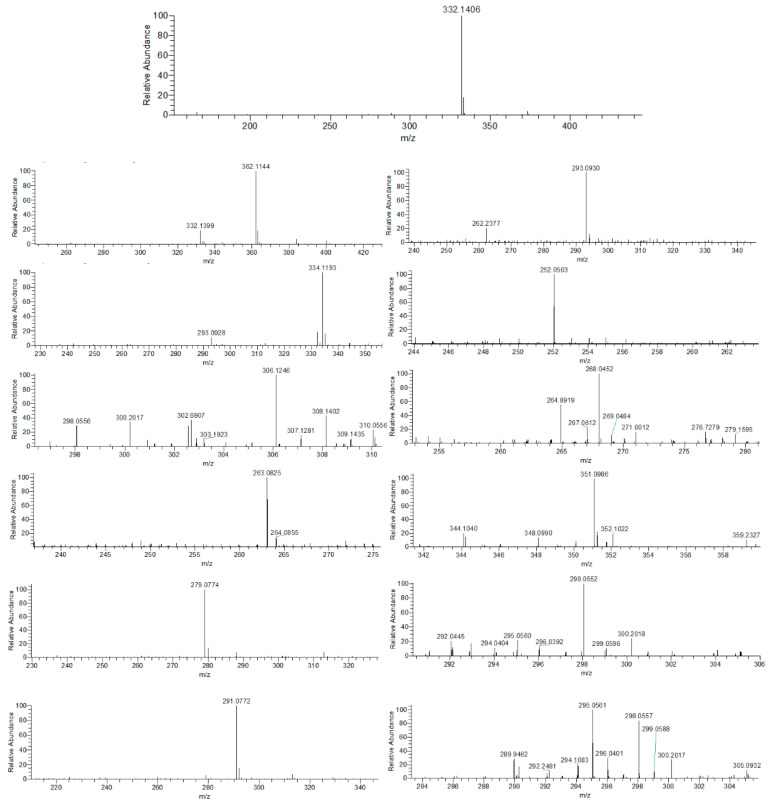
Photocatalytic degradation of CIP by g-C_3_N_4_/TCNQ/Eu in visible light.

**Figure 17:**
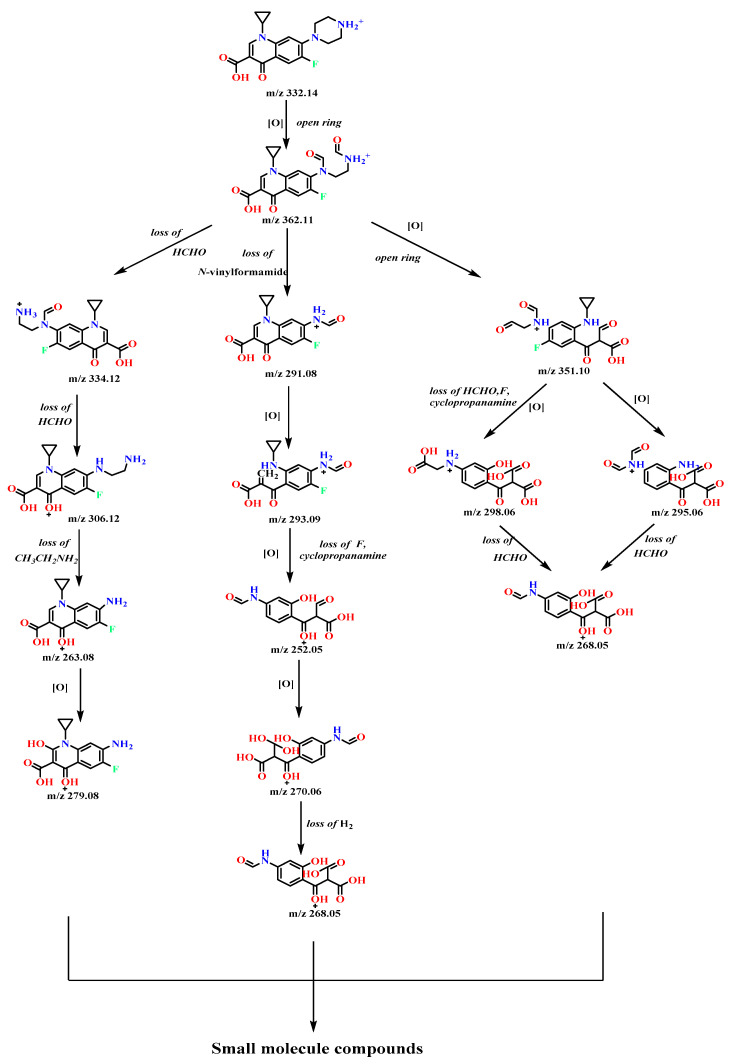
Possible pathway of g-C_3_N_4_/TCNQ/Eu photocatalytic degradation of CIP under visible light.

G-C_3_N_4_/TCNQ/Eu can mineralize CIP after visible light irradiation, which means that intermediate products are produced during degradation. In order to clarify the possible intermediate products converted by CIP during the degradation process, the results are shown in Figure 16 and Figure 17. The degradation products of the model compound ciprofloxacin at different stages were analyzed by electrospray electrostatic field orbital trap high resolution mass spectrometry (ESI-Q Orbitrap-MS) in the positive ion mode (ESI^+^). Combined with the obtained high resolution mass spectrometry data information, the structure of the degradation product was analyzed and confirmed. Based on the information of the product, it is speculated that the degradation path mainly passes through the following reaction channels.

The main degradation of the initial substrate ciprofloxacin (m/z 332.14) occurred in the groups “quinolone ring” and “piperazine ring”. Firstly, ciprofloxacin oxidized through the methylene group of piperazine ring, broke the C-C bond, and opened the ring to obtain the product m/z 362.11. Secondly, m/z 362.11 is degraded through three pathways. The first degradation pathway is to obtain products m/z 334.12 and m/z 306.12 through continuous removal of aldehyde groups after ring opening. The product m/z 306.12 was further deethylamined to yield product m/z 263.08, which was further hydroxylated by quinolone epoxidation to yield product m/z 279.08. The second degradation pathway was to deoxidize N-ethylformamide from m/z 362.11 to obtain product m/z 291.08, and further degradation showed that quinolone epoxidation and ring opening can be performed to obtain product m/z 293.09. M/z 293.09 defluorinated atoms and cyclopropylamine were oxidized again to obtain the product m/z 252.05, and then the oxidation of aldehyde groups was performed to obtain the product m/z 270.06, and then the product m/z 268.05 was obtained by oxidative dehydrogenation. The third degradation pathway is the deoxidation of N-methylformamide by m/z 362.11 and the ring opening of quinolone ring to obtain the product m/z 351.10, which is further degraded as deformaldehyde. The product m/z 298.06 was obtained by oxidizing and performing hydroxylation of the benzene ring with the fluorine atom and cypropyl ammonia, or the product m/z 295.06 was obtained by reoxidation and defluorination of the fluorine atom and cypropyl after deformaldehyde, and the product m/z 268.05 was obtained by oxidizing and hydroxylation of the aldehyde group and amino group. The products m/z 279.08 and m/z 268.05 may be degraded to small molecular compounds through quinolone ring opening, side chain removal, and further oxidation ring opening of a benzene ring.

### 3.7. Photocatalytic Mechanism Analysis

**Figure 18:**
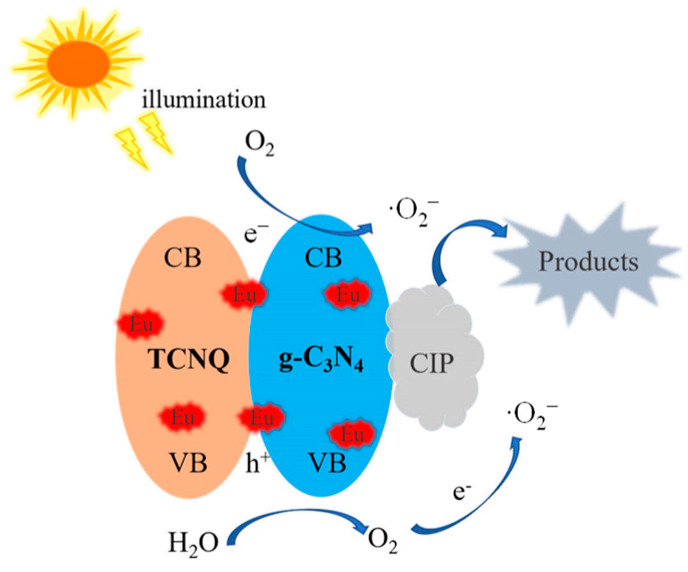
Possible CIP degradation mechanism.

Combined with the characterization analysis of the sample and the photocatalytic results, we speculate that the possible photocatalytic reaction mechanism of g-C_3_N_4_/TCNQ/Eu can be used to degrade CIP, as shown in Figure 18. CIP catalytic degradation is due to the effective absorption of visible light by g-C_3_N_4_/TCNQ/Eu and the formation of photogenerated electron holes under visible light irradiation, that is, g-C_3_N_4_/TCNQ/Eu + *hv*→e^−^ + h^+^. The REDOX potential at the bottom of the conduction band (CB) is more negative than that of O_2_/O_2_^−^, so the photogenerated electrons on the CB of the sample can react with O_2_ on the surface of the photocatalyst to form O_2_^−^, namely e^−^ + O_2_→O_2_^−^. On the other hand, h^+^ reacts with O_2_ and H_2_O to form a strong oxidizing agent OH [44,45]. The O_2_^−^ and OH produced in the system can directly react with pollutants, so CIP is catalyzed by the joint action of the two, that is, the product of O_2_^−^/·OH + CIP→products. In addition, TCNQ and Eu copolymerization modification can increase the specific surface area and pore volume of g-C_3_N_4_, enhance its adsorption capacity for degraded substances, and help to accelerate its catalytic degradation speed.

## 4. Conclusions

In this study, composite samples made of g-C_3_N_4_/TCNQ/Eu were effectively created and thoroughly described. The photocatalytic performance of CIP under the influence of visible light (>550 nm) was researched in order to assess the photocatalytic properties of the catalyst. Its cyclic stability, active species, intermediate product identification, and potential photocatalytic mechanism were disclosed. When the doping amount was g-C_3_N_4_/TCNQ/Eu-3, the photocatalytic performance of the g-C_3_N_4_/TCNQ/Eu composite sample was evaluated, and the D_CIP_ = 93.2% was the greatest. All samples for D_ENR_ adhere to the first-order kinetic equation when combined with the kinetic analysis of the photocatalytic process, and g-C_3_N_4_/TCNQ/Eu-3 has a higher k value of 0.0149 min^−1^, which is around 5 times that of g-C_3_N_4_. The g-C_3_N_4_/TCNQ/Eu composite sample shows improved reusability, as per the cycle stability test. The primary active species in the catalytic system of g-C_3_N_4_/TCNQ/Eu is O_2_^−^. The decomposition of CIP involves h^+^ as well. By using LC-MS to study two intermediates of the CIP degradation process, m/z 279.08 and m/z 268.05, their structures may be adequately inferred. Finally, based on the examination of intermediate by products and active species, the CIP breakdown mechanism was deduced.

## Figures and Tables

**Table 1:** Linear fitting data of photocatalytic reaction kinetics of CIP degradation in g-C_3_N_4_/TCNQ/Eu composite samples.

Sample Name	Regression Equation	*k*	*R* ^2^
g-C_3_N_4_	*y* = 0.0029*x* + 0.0885	0.0029	0.9598
g-C_3_N_4_/TCNQ/Eu-1	*y* = 0.0075*x* + 0.1109	0.0075	0.9668
g-C_3_N_4_/TCNQ/Eu-2	*y* = 0.0091*x* + 0.1455	0.0091	0.9641
g-C_3_N_4_/TCNQ/Eu-3	*y* = 0.0149*x* + 0.3297	0.0149	0.9513
g-C_3_N_4_/TCNQ/Eu-4	*y* = 0.0111*x* + 0.2290	0.0111	0.9796
g-C_3_N_4_/Eu	*y* = 0.0096*x* + 0.2290	0.0096	0.9518
g-C_3_N_4_/TCNQ	*y* = 0.0055*x* + 0.2143	0.0055	0.9578

## Data Availability

Data are contained within the article.
